# Educational program promoting regular physical exercise improves functional capacity and daily living physical activity in subjects with knee osteoarthritis

**DOI:** 10.1186/s12891-017-1912-7

**Published:** 2017-12-27

**Authors:** José Messias Rodrigues da Silva, Márcia Uchoa de Rezende, Tânia Carvalho Spada, Lucila da Silva Francisco, Fabiane Elize Sabine de Farias, Cleidnéia Aparecida Clemente da Silva, Claudia Helena de Azevedo Cernigoy, Júlia Maria D’Andréa Greve, Emmanuel Gomes Ciolac

**Affiliations:** 1 São Paulo State University (UNESP), School of Sciences, Campus Bauru, Physical Education Department, Exercise and Chronic Disease Research Laboratory, Bauru, Brazil; 2 University of São Paulo, School of Medicine, Department of Orthopedics and Traumatology, Sao Paulo, Brazil; 3 University of Guarulhos (UNG), Guarulhos, SP Brazil

**Keywords:** Daily living physical activity, Educational program, Physical fitness, Functional capacity, Physical exercise, Osteoarthritis

## Abstract

**Background:**

Physical exercise and educational programs promote several benefits for patients with knee osteoarthritis (OA). However, little is known about the effects of educational programs promoting the regular practice of physical exercise. The purpose of the present study was to assess the effect of an interdisciplinary educational program, emphasizing the recommendation for regular practice of physical exercise, on functional capacity and daily living physical activity in individuals with knee OA.

**Methods:**

Two hundred and thirty-nine individuals (50 men) with an established diagnosis of knee OA (degree I to IV in the Kelgreen and Lawrence scale) were randomly allocated into a multidisciplinary educational program (EDU; *n* = 112) or control group (CON; *n* = 127). Functional capacity (sit and reach, 6-min walking test (6MWT), timed up and down stairs test, timed up and go test (TUGT), and five times sit-to-stand test (FTSST)) and daily living physical activity (IPAQ, short version) were measured before, during (6 months) and after 12 months of follow-up.

**Results:**

Body mass index reduced significantly (*P* < 0.05) after 6 months, and remained reduced after 12-month of follow-up in EDU, but not in CON. EDU group improved (*P* < 0.05) timed up and down stairs (19%), TUGT (32.5%) and FTSST (30%) performance after 6 months of follow-up, which remained improved after 12 months of follow-up. Functional capacity did not change in CON, excepted for the timed up and down stairs performance that increased after 6 months (12%, *P* < 0.05), but returned to levels similar to baseline after 12 months of follow-up. There was also an increase (*P* < 0.05) in the prevalence of active and very active individuals, as well as a reduction (*P* < 0.05) in the prevalence of sedentary individuals in EDU group during follow-up. There were no significant changes on sit and reach and 6MWT performance during follow-up in both groups.

**Conclusions:**

The results suggest that an educational program emphasizing the recommendation for regular practice of physical exercise may be an effective tool for improving functional capacity and daily physical activity in individuals with knee OA.

**Trial registration:**

NCT 02335034, December 22, 2014.

## Background

The population aging is an unprecedented worldwide phenomenon that affects both developed and developing countries [1, 2]. The high prevalence of chronic disease that accompany the population aging, in association with the age-related deterioration that occurs in almost all physiological systems, results in reduced independence and quality of life in elderly individuals [2, 3]. Osteoarthritis (OA) of the knee is one of the most prevalent age-related chronic diseases, whereas one in four people over 55 years of age has persistent knee pain [4].

Although there is no curative treatment, the optimal management of knee OA requires pharmacological and non-pharmacological modalities, which are focused on alleviating disease symptoms, maintaining or improving patients’ functional independence and quality of life, and reducing disease progression to attempt to delay or avoid total knee arthroplasty [5]. The regular practice of physical exercise is an important non-pharmacological tool recommended for managing disease symptoms and improving functional capacity in patients with knee OA [3, 6, 7].

It has been suggested that educational programs may also have positive effects in individuals with knee OA [8]. For example, a study by our group showed that educational programs improved pain, function and quality of life (both physical and mental) after 6 [9] and 12 month of follow-up [10]. Therefore, to change the habits may have important clinical implication for this population, and the regular practice of physical exercise appears to be a crucial habit that should be incorporated in the daily living [3, 7, 9, 10].

The purpose of the present study was to assess the effect of an interdisciplinary educational program, emphasizing the recommendation for regular practice of physical exercise, on functional capacity and daily living physical activity in individuals with knee OA. We hypothesized that the educational program emphasizing the regular practice of physical exercise would be superior than conventional medical treatment.

## Methods

### Population and study design

The present investigation was a longitudinal prospective study conducted in a single center in Brazil that assessed 300 middle-aged and older patients with an established diagnosis of bilateral (294 patients) or unilateral knee OA for at least 1 year (Kellgren/Lawrence scale grades of I-IV) [11]. All volunteers were recruited from the Osteometabolic Disease Group, Institute of Orthopedics and Traumatology, School of Medicine, University of São Paulo, and they were under drug therapy (diacerein) for the 6 months prior to inclusion. To meet the eligibility criteria, patients needed to have met the American College of Rheumatology criteria for knee OA [12], to not have rheumatoid arthritis or any other rheumatologic disease other than OA, to be under routine care for knee OA for at least the previous 6 months, to not have any neurological problems, and to be able to understand and provide informed consent. Patients with uncontrolled cardiovascular or metabolic diseases, who underwent surgery or who had lower limb injuries during the previous 6 months were also not included. Baseline characteristics of the volunteers included in the present study are presented in Table 1.

**Table 1:** Baseline characteristics of the volunteers

Variable	EDU	CON
N	150	150
F/M	121/29	114/36
Age (years)	68.2 ± 9.6	66.3 ± 9.6
Body weight (kg)	78.0 ± 16.2	80.0 ± 15.1
Height (m)	1.58 ± 0.09	1.57 ± 0.08
BMI (kg/m^2^)	31.3 ± 5.9	32.1 ± 5.7
Bilateral osteoarthritis (N)	144	150
Unilateral osteoarthritis R/L (N)	2/4	0
Kellgren and Lawrence scale		
Degree I (N (%))	4 (2.7)	3 (2)
Degree II (N (%))	38 (25.3)	66 (44)
Degree III (N (%))	65 (43.3)	47 (31.3)
Degree IV (N (%))	43 (28.7)	34 (22.7)

*BMI* Body Mass Index, *CON* control group, *EDU* educational program group, *F* female, *L* Left, *M* male, *N* number of patients, *R* Right

**Fig. 1:**
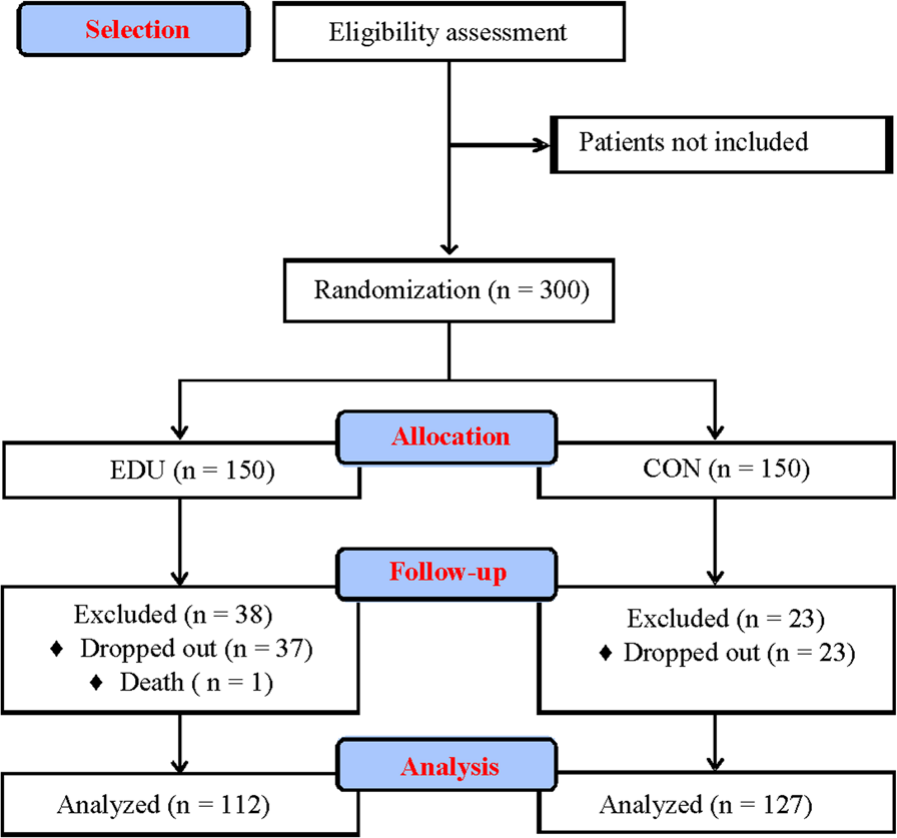
Schematic representation of the study design

Following screening for inclusion criteria, the volunteers have their functional capacity and physical activity levels assessed before, and after 6 and 12 months of follow-up. Volunteers were then randomly allocated into an educational program (EDU; *n* = 150) or control group (CON; n = 150) (Fig. 1). The EDU group participated in an educational program by a multidisciplinary group (orthopedic surgeons, physical education professionals, nutritionists, physiotherapists, occupational therapists, psychologists and social workers), which emphasized the recommendation for regular practice of home-based exercise in addition to the conventional clinical treatment. The CON group received only the conventional clinical treatment. The functional capacity and physical activity levels assessment were performed by members of the physical education team that were blinded for volunteers’ groups allocation.

The conventional treatment for knee OA included clinical follow-up by orthopedic surgeons; blood testing for metabolic syndrome (and referring for general practitioner clinical treatment as indicated), and calcium metabolism; X-rays, densitometry, and more specific image exams (e.g. ultrasound and magnetic resonance imaging) according to symptoms. Diacerhein was prescribed for all volunteers. A muscle relaxant and magnesium were prescribed for volunteers who reported cramps. All volunteers were oriented to take paracetamol and codeine when presenting moderate to severe knee pain. Low doses of nonsteroidal anti-inflamatory drugs were used only occasionally for severe pain, and during short periods of time. Vitamin D3 and calcium supplements were prescribed according to blood levels and bone densitometry results. Alendronate was prescribed for volunteers with osteoporosis. When necessary, orthotics (i.e. valgus or varus insoles, canes, walkers, and custom-made hand orthotics) were prescribed according to the disease severity, which was assessed by 3 orthopedic surgeons using the Kellgren and Lawrence classification, as previously described [10]. Volunteers with impaired mobility and pain were also referred to physical therapy and acupuncture.

### Functional capacity and physical activity assessment

All EDU and CON volunteers underwent functional capacity and physical activity levels assessment before and during 6 and 12 months of follow-up. Measures of functional capacity included the sit and reach, 6-min walking test (6MWT), timed up and down stairs test, timed up and go test (TUGT), and five times sit-to-stand test (FTSST), which were performed in a single day, following the sequence described below, and with a 3-min rest interval between them. The classic sit and reach test was used to assess the posterior thigh and lower back flexibility as previously described [13]. The 6MWT measured the distance covered while walking on a 15 m corridor without inclination. Volunteers were instructed to walk as fast as possible (without running) at a steady pace [14], and had their baseline (pre-test), peak (immediately after the end of the test) and recovery (2 min after the end of the test) heart rate measured with a digital frequency meter (Polar F1, Polar®, Finland). The timed up and down stairs test measured the time taken to ascend and descend a flight of 15 steps (15 cm high and 30 cm depth) as quickly as possible, starting from the standing position with their preferred leg, whereas it was allowed to use the handrail if necessary [15]. The FTSST measured the time taken to perform 5 repetitions of rising from a standard chair without armrest (seat height of 46 cm) to a full upright position as quickly as possible and without assistance [16]. The TUGT measured the time to get up from a chair without armrest (seat height of 46 cm) and without assistance, walk 3 m at normal speed, turn around, walking back and sitting down [17]. After the functional capacity assessment, the International Physical Activity Questionnaire (IPAQ) short version (validated in Brazilian population) was used to assess the daily physical activity level in all volunteers [18].

### Educational program

In addition to the conventional medical treatment, EDU group underwent an educational program with the health professional multidisciplinary team in order to make volunteers aware of their illness (OA) and their role on its treatment/management. The educational program occurred in a single day (Saturday, from 8 a.m. to 5 p.m.), and included 7 lectures of 30 min by each professional team, and 60-min workshops by the physical education, physical therapy and occupational therapy professionals, approaching the importance of their area in knee OA treatment/management.

The lectures occurred at morning. The orthopedic surgeon team introduced the anatomy of a joint and the pathophysiology of knee OA, its causes, irreversibility, and management. The physical education professionals lectured was about the health-related benefits of physical exercise and its role on knee OA management, the differences between physical activity and exercise, and the importance of improving physical fitness. The physical therapists also called attention for the role of performing physical exercise regularly, showed how to improve physical exercise to move from pain to a state of no pain, and talked about techniques to alleviate pain. The occupational therapists introduced the importance of protecting joints in daily activities by optimizing ergonomics and alternating different levels of energy expenditure. The nutritionist talked about the importance of a well-balanced diet (reduced quantity, colorful, whole grains, and low-calorie meals). The psychology team lectured about personality characteristics from childhood to adulthood, the difference between having a disease and being sick, the importance of their choices and not their conditions or feelings, and coping skills. The social work team showed where and how volunteers could and should include habits of regular leisure, sports and social gathering, and tasks. It is important to note that the role of changing habits was reinforced by each professional team, whereas the importance of practicing regular exercise was commented in all lectures.

The 3 workshops occurred at afternoon. The physical education workshop was about how to perform exercise (resistance and aerobic) at home, using low-cost alternative tools, as well as how to exercise at the appropriate exercise intensity. Volunteers were instructed to accumulate 30 to 60 min/day (bouts of at least 10 min) of moderate-intensity aerobic exercise (11 to 13 at the 6–20 rating of perceived (RPE) scale) [19], for 5 days a week. Alternatively to the moderate-intensity aerobic exercise recommendation, if physical capacity and disease severity allowed, volunteers were instructed to accumulate 20 to 30 min/day (bouts of at least 10 min) of vigorous-intensity aerobic exercise (14 to 16 at the 6–20 RPE scale) [19], for 3 days a week. Volunteers were instructed to perform the aerobic exercise modality they prefer (e.g.: walking, stationary cycling, dancing, swimming...). However, if weight bearing aerobic exercise was not tolerated (e.g.: there was a knee pain increase during or after exercise), they were instructed to perform stationary cycling or aquatic exercise. In addition to aerobic exercise recommendation, volunteers were instructed to perform 2 to 3 sets of 8 to 12 repetitions (1 to 2 min of interval between sets), in 10 resistance exercise (bench press, squat, seated row, knee extension, shoulder press, knee curl, triceps push-down, calf raise, biceps curl, and abdominal), for 3 times a week (nonconsecutive days). The volunteers were taught that they could perform the resistance exercises using weight-lifting machines, weight bearing, dumbbells, or some adapted material taught in the classes, (ankle weights, bars, elastic band, bottles with water or sand...), at an intensity between 14 and 17 at the 6–20 RPE scale) [19], using proper form and avoiding the Valsalva maneuver. The physical therapy team taught the volunteers how to adapt the exercise recommendation if they had knee pain and what they could do to alleviate knee pain at home, whereas the occupational therapy team taught how to protect the joints during both exercise and daily living activities.

The volunteers also received a printed material containing summaries of all lectures, and the exercises and practical recommendations of the 3 workshops, as well as a DVD with all the lectures and workshops. All volunteers were asked to watch the DVD and/or to read the booklet at least 3 times.

### Statistical analysis

The Kolmogorov-Smirnov test was used to analyze data normality. Parametric variables were reported as mean ± standard error, whereas non-parametric variables (physical activity levels) were reported as percentage of prevalence. Two-way ANOVA with repeated measures (group vs. time) was used to indicate inter- and intra-interventions differences in data presenting Gaussian distribution, and the Bonferroni’s post hoc test was performed to identify significant differences indicated by ANOVA. The Chi-square test was used to analyze inter- and intra-interventions differences in physical activity levels. The statistical program SPSS™ 17.0 for Windows (SPSS Inc., Chicago, IL, EUA) was used to perform the statistical analysis.

## Results

Two hundred and thirty nine volunteers completed the follow-up and were included in final analysis. The reasons for not inclusion in final analysis were no participation in the educational lectures and/or workshops (12 volunteers from EDU), no participation in all physical functional assessments during follow-up (25 volunteers from EDU and 23 volunteers from CON) and death (one volunteer from EDU) (Fig. 1). There were no significant differences in baseline characteristics between volunteers who completed the follow-up and those who did not (data not shown).

The two-way ANOVA indicated significant intra- and inter-intervention differences in body mass index (BMI) (*P* < 0.05). Post hoc analysis did not show significant BMI differences between group at baseline. However, BMI reduced after 6 months and remained lower after 12 months of follow-up (*P* < 0.05) only in EDU. With the reduction, BMI was significant lower (*P* < 0.05) in EDU than CON after 6 and 12 months of follow-up (Table 2).

**Table 2:** Body mass index before and during 6 and 12 months of follow-up

BMI	Before	6 months	12 months
EDU (*N* = 112)	31.1 ± 0.5	30.6 ± 0.4*^†^	30.5 ± 0.5*^†^
CON (*N* = 127)	32.1 ± 5.7	32.6 ± 6.3	32.6 ± 6.2

*BMI* Body mass index (kg/m^2^), *CON* control group, *EDU* educational program group. Asterisk denotes significant difference from before follow-up in the same group (*: *P* < 0.05). Dagger denotes significant difference from control group during the same period (^†^: *P* < 0.05)

There were no significant inter- or intra-intervention differences in 6MWT distance or HR response (Table 3). Although there was no significant intra-intervention difference, significant inter-intervention difference was found in sit and reach test (*F*_1, 42_ = 4.837, *P* = 0.033). Post hoc analysis showed that sit and reach levels were higher in EDU than CON throughout the follow-up (Table 4).

**Table 3:** Variables of 6-min walking test before and after 6 and 12 months of follow-up

	Variable	Pre	6 months	12 months
EDU (N = 112)	Distance (m)	368.6 ± 9.15	375.9 ± 9.8	385.6 ± 9.3
	HR resting (bpm)	76 ± 1.9	75.7 ± 2.5	77.8 ± 2.7
	HR peak (bpm)	97.2 ± 2.8	97.3 ± 3.5	99.2 ± 3.4
	HR recovery (bpm)	76.6 ± 2.3	75.9 ± 2.6	76.9 ± 2.7
CON (*N* = 127)	Distance (m)	357.5 ± 7.1	379.1 ± 7.6	366.1 ± 8.8
	HR resting (bpm)	75.0 ± 1.1	74.7 ± 1.2	74.9 ± 1.4
	HR peak (bpm)	103.6 ± 1.7	99.3 ± 1.7	98.3 ± 1.7
	HR recovery (bpm)	83.7 ± 1.0	77.0 ± 1.1	75.2 ± 1.3

*CON* control group, *EDU* educational program group, *HR* Heart rate

**Table 4:** Functional capacity before and during follow-up

	Variable	Before	6 months	12 months
EDU (*N* = 112)	Sit and reach (cm)	17.5 ± 0.8^†^	17.3 ± 0.9^†^	17.9 ± 0.9^†^
	Up and down stairs (sec)	30.3 ± 2.3	24.3 ± 2.4*^†^	24.4 ± 1.6*^†^
	TUGT (sec)	16.4 ± 0.7	11.5 ± 0.5**	10.4 ± 0.7**^†^
	FTSST (sec)	25.3 ± 1.3	17.1 ± 0.9***^††^	18.4 ± 1.1***^††^
CON (N = 127)	Sit and reach (cm)	12.0 ± 0.7	11.9 ± 0.8	13.2 ± 1.0
	Up and down stairs (sec)	34.5 ± 1.2	30.2 ± 1.3*	32.2 ± 1.9
	TUGT (sec)	12.4 ± 0.4	12.5 ± 0.4	12.6 ± 0.5
	FTSST (sec)	27.6 ± 1.3	24.9 ± 1.0	22.8 ± 1.4

*CON* control group, *EDU* educational program group, *FTSST* five times sit-to-stand test, *TUGT* timed up and go test. Asterisk denotes significant difference from before follow-up in the same group (*: *P* < 0.05; **: *P* < 0.01; ***: *P* < 0.001). Dagger denotes significant difference from control group during the same period (^†^: *P* < 0.05; ^††^: *P* < 0.01)

There were inter- (*F*_2.3, 94.4_ = 5.936, *P* = 0.002) and intra-intervention differences (*F*_1, 41_ = 6.006, *P* = 0.02) in up and down stairs test. Post hoc analysis showed that time to up and down stairs reduced nearly 19% (*P* < 0.05) after 6 months, and remained reduced (*P* < 0.05) after 12 months of follow-up in EDU volunteers. Time to up and down stairs also reduced during 6 months of follow-up (*P* < 0.05) in CON volunteers; however, this reduction was lower than that observed in EDU (19% vs. 12%, P < 0.05) and was not maintained after 12 months of follow-up (Table 4).

There was significant intra- and inter-intervention interaction in TUGT (*F*_2.7, 130.2_ = 6.568, *P* < 0.001). Post hoc analysis showed that TUGT improved (*P* < 0.01) after 6 months (nearly 30%) and remained improved after 12 months (nearly 37%) of follow-up only in EDU, which resulted in a TUGT level significant lower (*P* < 0.05) in EDU than CON after 12 months of follow-up (Table 4).

There were significant intra- (*F*_3, 153_ = 8.829, *P* < 0.001) and inter-intervention (*F*_1, 51_ = 8.956, *P* = 0.004) difference in FTSST. Post hoc analysis showed significant FTSST reduction (*P* < 0.001) after 6 months (nearly 32.5%) only in EDU volunteers, which were maintained after 12 months of follow-up. With this reduction, FTSST levels were significant lower (*P* < 0.01) in EDU than CON both after 6 and 12 months of follow-up (Table 4).

Daily physical activity levels are presented in Table 5. Lower prevalence (*P* < 0.05) of active and very active volunteers, as well as higher prevalence (*P* < 0.05) of sedentary volunteers were found in EDU than CON at before follow-up. However, the prevalence of active and very active volunteers increased (*P* < 0.05) after 6 months, and remained increased after 12 months of follow-up in EDU. EDU also reduced (*P* < 0.05) prevalence of sedentary volunteers after 6 months, which was maintained after 12 months of follow-up. A significant increase (*P* < 0.05) in the prevalence of very active volunteers after 6 months of follow-up, which was maintained after 12 months of follow-up, was the only change found in CON.

**Table 5:** Physical activity level before and during follow-up

	Classification	Before	6 months	12 months
EDU (N = 112)	Very Active (%)	2.5^†^	16*	18.5*
	Active (%)	21^†^	43*	42.5*
	Irregularly active A (%)	28.5	19	15.5
	Irregularly active B (%)	26	13	14.5
	Sedentary (%)	22^†^	9*	9*
CON (N = 127)	Very Active (%)	11.5	22*	25*
	Active (%)	42	38.5	39
	Irregularly active A (%)	16	15.5	18
	Irregularly active B (%)	18	18	11.5
	Sedentary (%)	12.5	6	6.5

*CON* control group, *EDU* educational program group. Asterisk denotes significant difference from before follow-up in the same group (*P < 0.05). Dagger denotes significant difference from control group during the same period (^†^: *P* < 0.05)

## Discussion

The primary finding of the present study was that EDU was superior to CON for improving several functional performance, including up and down stairs, TUGT and FTSST, in individuals with knee OA. According to our knowledge, the present study is pioneer in assessing the effect of an interdisciplinary educational program, emphasizing the recommendation for regular practice of physical exercise, on functional capacity and daily living physical activity in individuals with knee OA.

The effect of educational programs in individuals with OA has been previously investigated [20, 21]. Studies investigating the effects of educational programs associated with medical consultation have shown improvements in symptoms and in treatment compliance, which may have positive impact on quality of life and functional capacity in individuals with knee OA [20–22]. However, functional capacity has been primarily assessed through self-administered questionnaires [20, 23]. More recently, the TUGT and FTSST has shown to improve after educational programs in individuals with knee OA [24, 25]. The present study was pioneer in investigating the effect of an educational program on functional capacity measured through different tests that represent the ability to perform different daily activities. The improvement in TUGT, FTSST and up and down stairs performance observed in EDU, associated with the no changes found in CON, suggests that an educational program emphasizing the recommendation for regular practice of physical exercise can be effective for improving functional capacity in individuals with knee OA.

Although little is known about the effect of educational programs on functional capacity, it is known that physical exercise programs, specifically resistance training, promote improvement on functional capacity in individuals with knee OA [3, 7, 26]. For example, 13 weeks of a twice-weekly whole body resistance training program resulted in improvements on FTSST and stair climbing performance in individuals with total knee arthroplasty and OA in the contralateral knee [7], which were similar to those observed in the present study. The substantial improvements in FTSST (nearly 32.5%), TUGT (nearly 30%) and up and down stairs (19%) performance of EDU volunteers suggest that the educational program emphasizing the recommendation for regular practice of physical exercise used in the present study may be an alternative to conventional resistance exercise programs for improving functional capacity in individuals with knee OA.

It is possible that the substantial functional capacity improvements promoted by the educational program used in the present study may be due to its emphasis on the recommendation for regular practice of physical exercise. It is important to note that most studies analyzing the effect of educational programs in individuals with knee OA had only one or two health professionals providing the educational program [20–22, 27]. In the present study, the multidisciplinary educational program involved seven professional teams (orthopedic surgeons, physical education professionals, nutritionists, physiotherapists, occupational therapists, psychologists and social workers). The increase in the prevalence of active/very active volunteers and the reduction in the prevalence of sedentary volunteers observed in EDU during the follow-up suggest that the recommendation for regular practice of physical exercise may have an important role in the functional capacity improvements found in the present study.

The educational program also resulted in BMI reduction after 6 months and its maintenance after 12 months of follow-up, whereas CON group showed no changes in this variable. It has been shown that longevity and obesity results in worsening of knee OA symptoms and severity [27, 28], and that weight loss may improve pain, quality of life and functional scores in this population [29]. Thus, it is reasonable to suggest that the BMI reduction observed in EDU, although of small magnitude, may have influenced the improvement in functional capacity of those volunteers. In addition, BMI reduction may have been beneficial not only because of the reduction in joint overload, but also because of a possible improvement in obesity-related metabolic alterations [29]. Adipose tissue is a multifunctional organ that, in addition to its function as an energy store, plays an important role in endocrine system by producing adipokines. Several adipokines may act on immune system cells, leading to local and systemic inflammation [30]. In diseases with acute or chronic inflammation (i.e., such as knee OA), adipokines are associated with the pathophysiology of pain syndromes [31]. In contrast, physical exercise-related reduction in body fat is associated with prophylaxis of OA and pain improvement [31]. In addition to the OA-related benefits, the BMI reduction observed in EDU during follow-up may also have benefits for other chronic diseases present in the studied population, such as diabetes and arterial hypertension [32].

Functional walking capacity was measured by 6MWT, which can also be used as an indicator of aerobic capacity. This submaximal test has been widely used due its safety, feasibility, low cost and good tolerance, which allows its application even in advanced age groups, mainly because of its self-regulated pace and possibility of pausing or interrupting the walking if necessary [14]. It is suggested that the distance walked during the 6MWT should be compared with reference values, whereas predictive equations have been developed since 1998 [33]. In Brazilian population, the mean distance walked by adults aged 20 to 80 years is 566 ± 87 m and 538 ± 95 m for men and women, respectively [33]. For individuals with musculoskeletal limitations, it is suggested the use of a quadratic prediction model, which predicts that the population studied should walk 303.51 m during the 6MWT [33]. The EDU and CON volunteers walked 368.6 ± 9.15 m and 357.5 ± 7.1 m, respectively, during the 6MWT before follow-up. Thus, it is reasonable to suggest that volunteers initial aerobic capacity were above the predicted, which may be a reason why educational program was not effective for improving 6MWT performance. Supporting this hypothesis, a previous study assessing 6MWT performance in individuals with total knee arthroplasty and OA in the contralateral knee, that had a before follow-up 6MWT walking distance lower than the population in the present study (nearly 270 m), found substantial improvement (22.6%) in walking distance after 13 weeks of resistance training [7].

Although the sample size was large, the number of male volunteers was relatively small and may not represent the entire population of men with knee OA. Thus, future studies with greater prevalence of men are needed. The greater prevalence of sedentary individuals (22% vs. 12.5%) and lower prevalence of active (21% vs. 42%) and very active individuals (2.5% vs. 11.5%) in EDU than CON at baseline is also a limitation of present study, because it may interfere in the between group difference during follow-up. However, the within group analysis suggest that the educational program was effective for reducing the prevalence of sedentary individuals, and for increasing the prevalence of active and very active individuals during follow-up.

Reduced levels of functional capacity is associated with increased levels of disability, daily living dependence, hospitalization and mortality [7, 34, 35]. In this context, the functional capacity improvements observed in EDU individuals, but not in CON, suggest that the educational program of the present study may have important implications for reducing the risk of disability, daily living dependence, hospitalization and mortality.

## Conclusions

The results of the present study suggest that an educational program emphasizing the recommendation for regular practice of physical exercise was effective for improving functional capacity and daily physical activity in individuals with knee OA. However, the present educational program appears to be ineffective for improving aerobic capacity.

## Abbreviations

6MWT: 6-min walk test
BMI: Body mass index
CON: Control group
EDU: Educational program group
FTSST: Five times sit-to-stand test
IPAQ: International Physical Activity Questionnaire
OA: Osteoarthritis
TUGT: Timed up and go test
